# Supplementary material for: Implementation of GeneXpert MTB/Rif proficiency testing program: A Case of the Uganda national tuberculosis reference laboratory/supranational reference laboratory
Source: PLoS One. 2021 May 14;16(5):e0251691. doi: 10.1371/journal.pone.0251691 (PMC8121318; doi:10.1371/journal.pone.0251691)
Supplement: S1 Text — (PDF) [file pone.0251691.s001.pdf]

BCCM™ ITM	**CONFORMITY CERTIFICATE**	F025B
		12/04/2011
		Pagina 1 van 1

ITM NUMBER: M000995

SPECIES NAME: *M. fortuitum*

SPECIES RISK ASSESSMENT (ref. http://www.biosafety.be/RA/Class/ListBacteria.html)**:**

Pathogen class: 2

STRAIN INFORMATION:

The ATCC 19542 strain was obtained as *M. fortuitum minetti* by the Mycobacteriology Unit of the Institute of Tropical Medicine in 1967 and stored as freeze-dried material.

The freeze-dried material has been authorized for issue after checking for purity, viability and authenticity in 23/02/2011. Authenticity control was done by sequencing of the 16S rRNA gene.

DELIVERY CONDITIONS:

Storage at room temperature (15-30°C).

The control of viability, identity and mycobacterial purity on the batches for release, are performed according to the ISO 15189 standard (BELAC 147MED).
Accession, control, preservation, storage and supply of biological material and related information are performed according to the ISO 9001 standard.

BCCM/ITM uses the most optimal and up-to-date techniques for long term preservation of the strain, minimizing the risk of development of abnormalities or changes of the original features of the strain as the latter was deposited in BCCM/ITM. However, giving the biological nature of the material, BCCM/ITM declines any responsibilities related to changes of the characteristics of the strain.

OPTIMAL GROWTH CONDITIONS:

Medium: Löwenstein-Jenssen

Temperature: 30-38°C

Date: 07/03/2013 Signature of the curator

Received 13-Mar-2013
