# Supplementary material for: Implementation of GeneXpert MTB/Rif proficiency testing program: A Case of the Uganda national tuberculosis reference laboratory/supranational reference laboratory
Source: PLoS One. 2021 May 14;16(5):e0251691. doi: 10.1371/journal.pone.0251691 (PMC8121318; doi:10.1371/journal.pone.0251691)
Supplement: S2 Text — (PDF) [file pone.0251691.s002.pdf]

BCCM ITM	CONFORMITY CERTIFICATE	F025B<br>12/04/2011<br>Pagina 1 van 1

ITM NUMBER: 083715

SPECIES NAME: *M. tuberculosis*

SPECIES RISK ASSESSMENT (ref. http://www.biosafety.be/RA/Class/ListBacteria.html):

Pathogen class: 3

STRAIN INFORMATION:

ATCC 27294 obtained by the Mycobacteriology Unit of the Institute of Tropical Medicine in 2008 and stored as freeze-dried material since 2010.

The freeze-dried material has been authorized for issue after checking for purity, viability and authenticity in 05/07/2011. Identification of *M. tuberculosis* was done using DNA fingerprinting spoligo.

DELIVERY CONDITIONS:

Expiry date: 20 years after preparation, with storage at room temperature (15-30°C).

The control of viability, identity and mycobacterial purity on the batches for release, are performed according to the ISO 15189 standard (BELAC 147MED).
Accession, control, preservation, storage and supply of biological material and related information are performed according to the ISO 9001 standard.

BCCM/ITM uses the most optimal and up-to-date techniques for long term preservation of the strain, minimizing the risk of development of abnormalities or changes of the original features of the strain as the latter was deposited in BCCM/ITM. However, giving the biological nature of the material, BCCM/ITM declines any responsibilities related to changes of the characteristics of the strain.

OPTIMAL GROWTH CONDITIONS:

Medium: Löwenstein-Jenssen

Temperature: 35-37°C

Date: 07/03/2013

Signature of the curator

[signature]

Received 13-Mar-2013
[signature]
