# Supplementary material for: Implementation of GeneXpert MTB/Rif proficiency testing program: A Case of the Uganda national tuberculosis reference laboratory/supranational reference laboratory
Source: PLoS One. 2021 May 14;16(5):e0251691. doi: 10.1371/journal.pone.0251691 (PMC8121318; doi:10.1371/journal.pone.0251691)
Supplement: S3 Text — (PDF) [file pone.0251691.s003.pdf]

ITM NUMBER: 051626

SPECIES NAME: *M. tuberculosis complex*

SPECIES RISK ASSESSMENT (ref. http://www.biosafety.be/RA/Class/ListBacteria.html)**:**

Pathogen class: 3

STRAIN INFORMATION:

This strain was obtained from the SRL in Rome, Italy by the Mycobacteriology Unit of the Institute of Tropical Medicine in 2005 and stored at -70°C. It was mass-cultured in 2010 to serve on the panel for proficiency testing WHO/IUATLD round 17. Further aliquots were stored at -70°C.

The drug resistance profile of this mass-cultured strain has been characterized by the 28 participating supra-national TB reference laboratories globally as follows:

- Susceptible to isoniazid, ethambutol and ofloxacin (100% concordance), and to amikacin (90% concordance) and capreomycin (91% concordance)
- Resistant to rifampicin and streptomycin (100% concordance) and to kanamycin (92% concordance),

DNA sequencing has shown resistance conferring mutations in *rpsL* (Lys88Arg) and *rpoB* (Ser531Leu), but wildtype DNA in other resistance defining regions of *katG*, *inhA* and promotor, *rrs* (530, 915, 1400-1500), *embB* and *gyrA.*

CONDITIONS FOR USE:

ITM uses the most optimal and up-to-date techniques for long term preservation of the strain, minimizing the risk of development of abnormalities or changes of the original features of the strain. However, giving the biological nature of the material, ITM declines any responsibilities related to changes of the characteristics of the strain.

OPTIMAL GROWTH CONDITIONS:

Medium: Löwenstein-Jensen

Temperature: 35-37°C

INSTITUTE OF TROPICAL MEDICINE ANTWERP

Foundation of public utility
0410.057.701

Date: 7/3/2013

Signature of the coordinator of the rounds

Dr. Armand Van Deun
Mycobacteriology Unit
Institute of Tropical Medicine

Received 13-Mar-2013
[illegible]
