# Supplementary material for: Implementation of GeneXpert MTB/Rif proficiency testing program: A Case of the Uganda national tuberculosis reference laboratory/supranational reference laboratory
Source: PLoS One. 2021 May 14;16(5):e0251691. doi: 10.1371/journal.pone.0251691 (PMC8121318; doi:10.1371/journal.pone.0251691)
Supplement: S1 File — (PDF) [file pone.0251691.s004.pdf]

The Republic of Uganda

MINISTRY OF HEALTH

NATIONAL TUBERCULOSIS AND LEPROSY CONTROL PROGRAMME

Proficiency Testing Plan

SOP PT008 **Version 3.0** **Effective date:** **Initials authorizer:**

SOP Approval

	Name	Signature	Date
Prepared by			
Reviewed by			
Authorized by			
Date Retired:			

Approved changes

Brief description of the change

Annual Changes and Reviews

Name of reviser	Changes compared to previous version

1. Proficiency Testing Plan

2. Objectives and scope

This SOP describes the proficiency testing schemes plan for microscopy, culture, genotypic and phenotypic susceptibility testing. This SOP is applicable to all staff handling the PT scheme activities.

3. Abbreviations, definitions and terms

DST – Drug Susceptibility Testing
EQA – External Quality Assessment
LPA – Line Probe Assay
LJ – Lowenstein Jensen
NTRL – National TB Reference Laboratory
PT – Proficiency testing
SOP – Standard Operating procedure
TAT – Turn Around Time
ZN - Ziehl Neelsen

Quantitative scheme — where the objective is to quantify one or more measurands of the proficiency test item;
Qualitative scheme — where the objective is to identify or describe one or more characteristics of the proficiency test item. The results of qualitative tests are descriptive and reported on a categorical or ordinal scale, e.g. identity of micro-organisms, or by identification of the presence of a specific measurand (such as a drug or a grading of a characteristic).
Simultaneous scheme — where proficiency test items are distributed for concurrent testing or measurement within a defined time period;
Continuous scheme — where proficiency test items are provided at regular intervals;

4. Tasks, responsibilities and accountabilities

Task	Responsible	Accountable
Preparation of PT plan	PT scheme manager	Lab manager

5. Safety and environment

N/A

6. Procedure

6.1 Scheme Scope

The NTRL PT schemes covers microscopy which caters for both Ziehl Neelsen (ZN) and florescence microscopy (FM), genotypic DST which caters for LPA, GeneXpert and any other molecular based TB diagnostic assays, Phenotypic Drug susceptibility testing (DST) which caters for both solid and liquid culture and their respective DST methods both first and second line DST. This is a qualitative and semi qualitative, continuous PT scheme and simultaneous PT scheme

6.2 Schedule

EQA panels category	GeneXpert
Frequency/ year	Biannual
Sending out Month	February and August
Quantity	04 isolates/round
Expected TAT (from date of shipment)	44 days

6.3 Design of proficiency testing scheme

6.3.1 Planning

The National TB Reference Laboratory (NTRL) will identify and plan for processes which directly affect the quality of the proficiency testing scheme and ensure that they are carried out in accordance with prescribed procedures as well as involving the stakeholders' interests and relevant information.

1. **Objective of the PT scheme**
   The objective of the PT scheme is to provide PT items to participants to reinforces their quality assurance systems and provides the basis for any corrective action in case of non-satisfactory performance.

2. **Purpose of the PT scheme**
   The purpose of the PT proficiency testing (PT) scheme is to check the competence of participating laboratories by a statistical evaluation of the data they obtain on analyzing centrally distributed materials.

3. **Basic design of the PT scheme**
   The design of the NTRL PT scheme includes randomly selected sub-samples from a bulk homogeneous supply of material which are distributed simultaneously to participating laboratories.

4. **The name and address of the proficiency testing provider;**

National TB Reference Laboratory Uganda
P.o.Box 16041
Kampala Uganda
Kampala Uganda
Plot 106-1062, Butabika Road, Luzira.
Opposite Butabika Hospital
Toll free: 0800111133
Email: pt@ntrl.or.ug

5. The name, address and affiliation of the coordinator and other personnel involved in the design and operation of the proficiency testing scheme;

Joel Kabugo
PT Scheme Coordinator – GeneXpert MTB/RIF PT Scheme
National TB Reference Laboratory Uganda
P.o.Box 16041, Kampala Uganda
Plot 106-1062, Butabika Road, Luzira.
Opposite Butabika Hospital
Toll free: 0800111133
Tel: +256 704 342151 Email: jkabugo@ntrl.or.ug

6.0 Review of requests, Tenders and contracts

Review of requests tenders and contracts will be done annually or in the event that any of the parties wishes t ammend the contracts or tenders.
The following procedures will be followed;
The review will include all aspects of the contracts or tenders and requests. The review will assess the procedures documented in the initial aggrement that will be followed during the event of breach of contract of review of the aggrement.
The ammendments to be made will be communicated to all the affected parties such that appropriate measures are instituted. Records of such reviews will be maintained by the quality manager

Sub contracting

NTRL-Uganda, the PT service provider, will not sub contract any operations of PT scheme preparation, package and result analysis etc. The PT provider contracts an IATA approved company for courier/POSTA services of PT items and results. In instances, where preparation of PT panel is sub contracted to another PT provider, NTRL will take full responsibility of the outcome including analysis of results and dissemination of feedback reports

6.1 Confidentiality Arrangement

The NTRL has put in place measures to ensure that the PT items,results among others are as confidential as possible according to SOP P065 Confidentiality.

Confidentiality of results

All participant information including details, results, performance among others is treated confidential by the PT provider unless otherwise stated.
Waiver of confidentiality forms are distributed to new particpants together with the PT scheme enrollment forms.

6.2 Criteria to be met for participation

The NTRL will ensure that each lab that participates in this PT scheme meets the minimum standards. Each willing participant will communicate to the NTRL-Uganda scheme Manager about the willingness to participate in writing. Other forms of contracts like SRL links and agreements also apply. The communication can be in form of email or hardcopy. All forms of communications will be proof of the particpantion and request for enrollment in the PT scheme.

Participants within Uganda:
The Uganda NTRL is mandated by the Ministry of Health to ensure that TB laboratories in the country produce reliable and accurate results. Therfore, NTRL provide profciency testing materials to all laboratories within Uganda. The Laboratory enrollment codes will use codes o of the National microscopy blinded rechecking alreading ongoing in the country and only add Suffix at the begining i.e. GXP – GeneXpert MTB/RIF® assay PT, DST e.g GXP 802-03 for a laboratory in GULU TB region (8), gulu district (02) and (laboratory code (-03) .

Enrollment procedure
All participants who are not part of the Uganda National laboratory netowrk/NTLP and Ministry of Health will be reguired to enrol in the Ugand PT scheme.

Steps for enrollment

1. Write formally to NTRL PT scheme manager or representative.
2. Fill in Enrollment Questionnaire Form PT 008 F1
3. Fill in **'Participant Biosafety Compliance Letter agreement between PT provider-Uganda NTRL and PT Participant (PT 008 F3) for** laboratories to participate in PT scheme involving manipulation of *Mycobacterium tuberculosis* live organisms
    a. NB: Should be submitted by 31 March of every new year for existing particpants
4. PT provider sends enrollment certificate with number to participanting lab. The certificate has the enrolment number which is the same as the laboratory/participant code for the PT schemes.
5. Add and/or update new and existing laboratory participant(s) details/contents to excel ' PT 008 F4 Participant enrolment log" *if applicable*
6. NB: Steps (iii) is on annual basis
7. In addition, once a particpant confirms participation and is approved by PT scheme manager add details on PT 008 F5 Annual Participation Log.
    a. NB. Only participants on the PT 008 F5 "Annual Participation Log" will receive PT panels in that particular year.

Assigning participant laboratory number (for participants out of the Uganda NTLP/MOH laboratory network)

1. The participant laboratory number will be assigned by the PT scheme manager or delegatee
2. The number will consist of the PT category sufficx and a three digit number e.g. GXP -001, MIC-001, DST-001 where GXP is GeneXpert MTB/RIF(R) assay, MIC, Microscopy PT and DST – culture and DST PT, 001 is the number of the particpant assigned in ascending order on the particpant log form.
3. Each participant receives one 'three digit number" and only the suffix i.e.GXP, MIC, DST changes accordingly.
4. The three digit number (e.g. 001) assigned to a participant 1 shall not be assigned to any other participant e.g particpant 2 even if the former (participant 1) no longer receives panels from the Uganda NTRL PT scheme.

Costs

1. Participants under the Uganda NTRL/NTLP-Ministry of Health laboratory network: No cost attached.
2. Participants under the Regional grants between Global Fund and ECSA HC-Uganda grant: No costs attached.
3. Other participants: There is costs attached to each PT scheme and provided upon request.

6.3 Selection of the measurand(s) or characteristic(s) of interest, including information on what the participants are to identify, measure, or test for in the specific proficiency testing round;

The NTRL will clearly identify the measurand, and chracteristics of interest and these will be indicated on the "Participants' instructions manual" for each PT scheme. The selection of the measurand(s) and characteristic(s) for each PT scheme will depend on international Procedures and guidelines such as WHO, IUATLD etc.

6.4 Potential major sources of errors involved in the area of PT offered;

The NTRL re-affirms that the processes of PT item preparaton right from planning through preparation to result analysis will be quality assured to minimise sources of errors for each PT item

Potential sources of errors for each PT item and ways of minimising them are identified under the respective PT SOPs.

6.5 Requirements for the production, quality control, storage and distribution of PT items;

The NTRL Management is committed to providing the necessary requirements for the PT scheme in terms of trained and competent staff, quality control procedures, materials and procedures for storage and distribution. (See SOP PT009 Preparation of PT items and SOP PT012 Handling and storage of PT items)

6.6 Precautions to prevent collusion between participants or falsification of results, and procedures to be employed if collusion or falsification of results is suspected

The NTRL has put measures to prevent collusion of participants and falsification of results such as homogeneity and stability during planning and preparation, intructions to participants, labelling of PT material. In the even that collusion or falsification of results is suspected then the NTRL will confirm with the participants this occurance and corrective measures will be instituted; these measures may include cancellation of the results analysis of that specific participant/ round or the issue of a new panel all together to the affected participant.

6.7 Information to participants and schedule for the various phases of the PT scheme;

The NTRL has put in procedures and instructions to particpants including the different PT schemes and schedules. These instructions will either be electronically disseminated to the participants or a hard copy may also be sent together with the PT materials. (See SOP PT011 Instruction to participants)

6.8 Frequency and dates upon which proficiency test items are to be distributed to participants, the deadlines for the return of results by participants and, where appropriate, the dates on which testing or measurement is to be carried out by participants;

In the table below is the frequency of providing PT material and procedures for reporting of results.

Summary of PT schemes and reporting schedules

A	B	C	D	E	F	G	H
PT Scheme	PT schedule	Total days from Dispatch to Closing date	Prepare Individual and summary report (from closing date)	Present to PT team (Days from D)	Dispatch Individual and Summary report (Days from E)	Total	Total Months
GeneXpert	02 (Feb and Aug)	44	21	7	14	86	2.9

6.9 Information on methods or procedures which participants need to use to prepare the test material and perform the tests or measurements;

Instructions to participants will be provided for each PT scheme to particpants before testing of the PT items begin. (See SOP PT011 Instruction to participants)

6.10 Procedures for the test or measurement methods to be used for the homogeneity and stability testing of proficiency test items and, where applicable, to determine their biological viability;

The NTRL have provided procedures and materials for ensuring homogeneity and stability of the PT material. (SOP PT009 Preparation of PT items)

6.11 Preparation of any standardized reporting formats to be used by participants;

All participating labs are provided with standard reporting forms, formats and contacts for each PT scheme. (See SOP PT015 Reporting of PT results)

6.12 Detailed description of the statistical analysis to be used;

All PT test results will be analysed according to international stardards using the apropriate statistical analysis procedures and where applicable have been validated. The assigned value is determined by the NTRL; the PT provider. (See SOP PT010 Statistical design and assigned values)

6.13 Origin, metrological traceability and measurement uncertainty of any assigned values;

All equipment, materials and procedures used in the PT schemes have been verified/validated. All procedures invloving the assigned value have been documented and can be traced .
(See SOP PT010 Statistical design and assigned values)

6.14 Criteria for the evaluation of performance of participants;

The NTRL has developed a procedure of evaluating performance of participants which will involve the review of performance from all or the majority of the participants and analysed using the statistical package.

6.15 **Description of the extent to which participant results, and the conclusions that will be based on the outcome of the proficiency testing scheme, are to be made public;** Performance of participating labs and the oconclusion of the outcome of the PT scheme will be reported to the contacts of that lab and others in that round of the PT for purposes of comparision. However, all reports to the public will not bear names of the particpant but only codes.

6.16 **Procedure actions to be taken in the case of lost or damaged proficiency test items;**

If the participant provides substancial proof of loss or damage, then this will not be inculded in the analysis. Where possible an emergency PT panel will be sent to the participant.

6.17 Procedure of appealing against PT scheme results/reports by participants

Participants are free to appeal to the scheme manager any results/ reports for any round of PT that they deem un-satisfactory or un re-representative of their competance or work. The NTRL will then review the the appeal to for the appropriate feedback/ corrective action and *where applicable* amended reports and results will be provided.

6.18 Advisory services

Advisory services at the Uganda NTRL PT schemes is provided internally by the Proficiency testing team (PT team) that comprises of NTRL staff with the necessary technical expertise and experience in the relevant field of testing, calibration, sampling or inspection, as well as statistics for PT scheme analysis and reporting. Where it is required, the Uganda NTRL will seek for external advisory services from the SNRL network coordinating laboratory of Institute of

Tropical Medicine (ITM), Antwerp or any other as seen fit by the PT team. Quorum for a meeting is 5 members

7. Related forms

N/A

8. References

17043 standard
