# Supplementary material for: Implementation of GeneXpert MTB/Rif proficiency testing program: A Case of the Uganda national tuberculosis reference laboratory/supranational reference laboratory
Source: PLoS One. 2021 May 14;16(5):e0251691. doi: 10.1371/journal.pone.0251691 (PMC8121318; doi:10.1371/journal.pone.0251691)
Supplement: S2 File — (PDF) [file pone.0251691.s005.pdf]

SOP Approval

	Name	Signature	Date
Prepared by			
Reviewed by			
Authorized by			
Date Retired:			

Approved changes

Brief description of the change

Annual Changes and Reviews

Name of reviser	Changes compared to previous version

1. Preparation of Proficiency Testing Items

2. Objectives and scope

This SOP describes the preparation of proficiency testing items for microscopy, culture, genotypic and phenotypic susceptibility testing schemes. This SOP is applicable to all staff handling the PT scheme activities.

3. Abbreviations, definitions and terms

AFB – Acid Fast Bacilli
DST – Drug Susceptibility Testing
EQA – External Quality Assessment
IUATLD – International Union against Tuberculosis Lung Disease
LJ – Lowenstein Jensen
NaOH – Sodium Hydroxide
NTRL – National TB Reference Laboratory
PT – Proficiency testing
TAT – Turn Around Time

4. Tasks, responsibilities and accountabilities

Task	Responsible	Accountable
Preparation of PT material	Scheme Manager and coordinators	Lab manager

5. Safety and environment

Treat all specimens as potentially infectious. Work in biological safety cabinet *unless otherwise*.

6. Procedure

6.1 Materials needed

- Standard control strains of *Mycobacterium tuberculosis (MTB)* H37R*v* , RS, and *M.fortitium* well characterised strains from the Uganda NTRL/SRL
- Phosphate buffered saline (PBS) or sterile water for the Negative panels.
- Universal bottles
- Plastic loops
- Sterile water (distilled and autoclaved)
- Beads
- Autoclave
- Cryovials

6.2 Source

1. Standard control strains of *Mycobacterium tuberculosis (MTB)* H37R*v* , RS, and *M.fortitium* well characterised strains from the Uganda NTRL/SRL database
2. MTB strains with know resistant pattern for RIF and INH (through molecular typing either done in Antwerp SRL or at NTRL Uganda) will be used for preparation of all LPA panels.
1. Susceptible strains to both RIF to be used will be standard strain of H37R*v* or other well chararcterised available at the Uganda NTRL.
2. Phosphate buffered saline (PBS) for the Negative panels.
3. Record selected strains on (use *PT 009 F7: Source of strains for culture and DST)*
   a. ***NB:*** Preparation of strains for should begin at least 3 months to allow for adequate time for LJ culture
      i. culture of selected strains on LJ for maximum of 3 weeks and culture of the heat killed strains on MGIT to check for viability check

6.2.1 Preparation procedure

Liquid panels (use of cryovials)

1. Strain suspensions of 1.0 Mcfarland standard are prepared from Solid culture
2. Autoclave the strain suspension
3. Set liquid culture using the autoclaved strain suspension. Negative results on culture show proof of inactivation.
4. Dilute the bacterial suspension by; preparing 0.5 Mcfarland standard ($1.5x10^8$bacilli/mL):
   a. aliquote 0.5 mL of 0.5 Mcfarland standard and add to 9.5 mLs of sterile water to obtain a bacterial concentration of 10^6 bacilli/mL.
   b. Using the concentration of 10^6 bacilli/mL, aliquot 0.5 mL and add to 9.5 mL of sterile water to obtain a concentration of 10^4 bacilli/mL which shall be used for making Postive panel numbers .
   c. **NB.** The volume of bacterial concentration of 10^4 bacilli/mL to prepare shall vary with the number of panels to be supplied.
5. Aliquot 01 mL of bacterial concentration of 10^4 bacilli/mL into croyvials to make the postive panels
6. Like wise, for the negative panels aliquote 01 mL of PBS or sterile water into cryovails. Four (4) PT cryovials will consitute a panel.
7. Ensure that cryovials from the same batch are put in one prelabelled big ziplock bag.
8. These represent the PT items ready for packaging and labelling for each individual particpant laboratory

Quality control and validation

1. Precaution should be taken to avoid cross-contamination. ***Always prepare negative panesl first.***
2. Pick randomly 5 cryovials per PT strain and and test on GeneXpert MTB/RIF assay
3. Results should be 100% of the Expected/target PT results

Note carefully;

1. Ensure that PT items i.e. cryovials from the same batch representing the same strain are packed in one ziplock bag or cryo-box which is clearly labelled with strain ID, PT scheme Name, Round number and Year.

2. These are cryovials from which individual PT items are picked are picked for each individual participanting lab.
3. **NO CRYOVIALS FROM DIFFERENT STRAINS SHOULD BE PUT IN THE SAME ZIPLOCK BAG OR CRYO BOX** to avoid mix up and cross contamination

6.2.2 Labelling and Packaging of Culture and DST PT items

See SOP PT 013 Packaging, labelling and distribution of PT items

6.2.3 Homogeneity and Stability

(a) Homogeneity

1. The laboratory shall ensure the preparation procedure above is strictly followed to ensure homogeneity

Procedure for ensuring homogeneity

- Potential Isolates/strains are stored at -80ºC to minimise genetic changes
- Number of subcultures is limited to 3 -6 times
- Isolates are selected from strains that had good concordance (at least 80%) in the SRLN rounds
- Sub culture of isolates/strains on LJ culture and selecting those showing those with easiest growing organsims
- Use of sterile beads allows breakdown of colonies to get a homogenous suspension
- Preparation of McFarland 1.0 standard from the postive LJ slant and use of cell culture flasks
- Several sterility checks in the procedure above allow to discard any strain contaminated

Measurement of homogeneity

1. The QC/validation tests done above are taken as the measure of homogeneity.
2. For homogeneity to pass, 100% performance must be obtained.

(b) Stability

Procedure for ensuring stability during transportation

- Use of IATA triple packaging ensures no breakage of materials
-

Measurement of stability

The PT panel to be tested for stability must mimic the transport conditions of the dispatched panels as much as possible i.e. packaged and left at room temperature

One PT panel retained at NTRL is tested at least two weeks by doing GeneXpert MTB/RIF® analysis.
The GeneXpert MTB/RIF® results must score 100% of expected results on both INH and RIF drugs.

6.2.4 Storage and transport

See SOP PT 012 Handling and Storage of PT items.

6.2.5 Retaining of PT items

- Keep all properly labelled isolates per round (at least two (02) PTs) as indicated above in slide box
- Label one as EXCESS 1 and the other EXCESS 2
- Label cryobox as EXCESS and round number
- Keep cryo box at -80^{0}C

- Retain the PT slides until all the results of the participating labs have been received, reports analyzed and feedback reported or before the next round begins

Retaining

1. Keep all properly labelled isolates per round (at least two (02) PTs) as indicated above in slide box
2. Label one as EXCESS 1 and the other EXCESS 2
3. Label slide box as EXCESS and round number
4. Keep slide box at -80^{0}C
5. Retain the PT slides until all the results of the participating labs have been received, reports analyzed and feedback reported or before the next round begins

All procedures starting from planning through the preparation upto end of analysis of PT results and reporting is tracked using different PT schemes tracking form;

Handling and storage of GeneXpert (Liquid method)

1. Retrieve selected strains from -80°C freezers.
2. Thaw at room temperature
3. Use Quality controlled LJ culture slants and for subculture
4. Keep stocks of autoclaved strain suspension of the PT material at -80°C.
5. Properly label with Name of PT, ID of strain, Round number of PT material.
6. PT items transferred to cryovials for a particular participating are stored in Ziplock bags properly labeled with Name of Lab, PT name and PT Round Number.
7. Stores those pending shipment at -20°C
8. Keep the properly labeled excess cryovials of the PT material in cryoboxes and store at -80°C freezer.
9. Discard all the PT materials related to this round after results and reports have been finalized.
10. A portion of the PT scheme is then stored in 7H9 media containing glycerol and kept at -80°C and respective location of the isolates captured in the storage database respectively.

7. Related documents

GeneXpert Assay

8. Related forms

P031 F2 Pasteur Pipette Calibration Log

9. References

N/A
