# Supplementary material for: Implementation of GeneXpert MTB/Rif proficiency testing program: A Case of the Uganda national tuberculosis reference laboratory/supranational reference laboratory
Source: PLoS One. 2021 May 14;16(5):e0251691. doi: 10.1371/journal.pone.0251691 (PMC8121318; doi:10.1371/journal.pone.0251691)
Supplement: S3 File — (PDF) [file pone.0251691.s006.pdf]

SOP Approval

	Name	Signature	Date
Prepared by			
Reviewed by			
Authorized by			
Date Retired:			

**Approved changes**
Brief description of the change

**Annual Changes and Reviews**	
Name of reviser	Changes compared to previous version

1. Packaging, labelling and distribution of Proficiency Testing Items

2. Objectives and scope

This SOP describes the proficiency testing items are packaged, labelled and distributed to the proficiency testing scheme participants. This SOP is applicable to all staff handling the PT scheme activities.

3. Abbreviations, definitions and terms

AFB – Acid Fast Bacilli
DST – Drug Susceptibility Testing
EQA – External Quality Assessment
IATA – International Air Transport Association
IUATLD – International Union Against Tuberculosis Lung Disease
LJ – Lowenstein Jensen
NaOH – Sodium Hydroxide
NTRL – National TB Reference Laboratory
PT – Proficiency testing
TAT – Turn Around Time

4. Tasks, responsibilities and accountabilities

Task	Responsible	Accountable
Packaging, labelling and distribution of PT items	All lab personnel	Lab manager

5. Safety and environment

Treat all specimens are potentially infectious. Work in biological safety cabinet *where applicable*.

6. Procedure of packaging, labelling and distribution of PT items

NB. Labelling and Packaging is mostly based on local and international regulations such as International Air Transport Association (IATA)

6.1 Labelling and packaging of GeneXpert PT items

6.4.1 Liquid samples

1. Each participating lab receives 04 PT items Q1, Q2, Q3 and Q4 for GeneXpert MTB/RIF® PT.
2. Cryovials will be labelled Q1….to Q4 with GXP for GeneXpert, round number and year when the round is being sent out.
3. Print adhesive tube labels as mentioned in (2) above
   NB: Ensure you print adequate labels according to the number of participants you are going to send to.
4. Label four medium sized biohazard bags Q1 to Q4 to which each respective batch of item will be placed prior to packaging into individual ziplock bags that are sent to participants.
5. Labelling one item at ago, pick one cryovial at a time from each batch of cryobox and pack each into a small ziplock bag and place each batch into the above label biohazard

bag. Repeat this until a sufficient number of items enough to supply all sites and for retaining has been achieved.
NB. Caution should be taken to avoid mix up of different batches; preferably work alone or at times when there is no much distraction.

6. Another personnel should confirm that all tubes are appropriately labelled before transferring to a big ziplock bag.
7. Transfer 04 individually packed tubes to big Ziplock bag, clearly label the ziplock bag with the code of the participating lab, PT round and Year using adhesive labels
8. Repeat step (7) for PT panel of second participating lab
9. Store the ziplock bags tightly closed at 4°C waiting for shipment.
(NB. Since this is no infectious, hence use transport box same as for microscopy PT)
10. Using microsoft word, print out details of the consignee and consigner (This list is updated whenever there are new participants by the scheme manager or delegate)
    (a) (*NB: cut the print out to fit on the size of the space allocated on the transport box both for consignee and consigner details)*
    (b) *Consignee and consigner details should at least include names of contact person, address, e-mail (if available) and phone contact(s) e.g.*

CONSIGNEE
Lab Contact person: XXXXXXXX
Position of contact person: xxxxxx
Lab Name: xxxxxxx
Physical address: xxxxxxxxxxxxx
Email:xxxxx
Tel:xxxxx
Mobile:xxx

CONSIGNER
EQA scheme Manager Name:xxxxxx
National TB Reference Laboratory
P.o. Box 16041, Kampala Uganda
Plot 1062-106, Butabika. Uganda
Email: for scheme manager. Toll free: 0800111133
Mob: For scheme Manager

11. Print and cut out "Temperature Label _

Packed and Transported at:
Ambient **Temperature**

1. When the courier company confirms date and time of pick (*NB. sometime PT items are delivered by an individual)*
2. Then proceed to transfer zip lock bags from (vii) above into each pre-labeled transport box. *NB; Each participant receives one transport box*
3. Insert instruction and result worksheet into an envelope and transfer it into the transport box.
4. Another laboratory tech confirms the labelling and packaging has been done right by
    (a) Crosschecking if each participant pack has 04 cryovials or tubes.
    (b) Crosschecking each cryovial/tube is labelled
    (c) Cross checking if all reserve tubes and cryoboxes are properly labelled.
    (d) Package of Isolates for stability testing are kept after, second person has confirmed the packaging, Put "safety seal". Alert participants not open the safety container (secondary packaging)" if seal is tampered with.

In-case of more than one PT in the same package

1. Incase more than one participant reside in the same location such as building or country, PT panels for more than one participant are packed in the same transport box.
2. In this case, the PT panels for each participant are packed separately in a ziplock and clearly labeled with name of participant, code and Round Number
3. Notify all the participants before shipment.

6.2 Distribution: Shipment and transportation of PT panels

6.2.1 Time schedule and notifications

1. Notify contracted courier company about an upcoming shipment and in addition notify participants
   (a) Courier company
   - PT scheme manager or delegate notifies NTRL procurement/administration team to solicit and contract a courier company to ship PT items. This is done at least two weeks before the expected date of shipment
   - PT scheme manager or delegate provides addresses of the PT participants so as to prepare the necessary paper work required (*this can be country specific hence the need for the courier company to advise where applicable)*

   (b) International participants (out of Uganda)
   - The PT scheme Manager or delegate notifies each participant lab separately through email to the contact person about a PT panel to be shipped. This is done one **(01) week** before the expected date of shipment.
     Information in the email body should at least include; date expected to ship details on PT panel, number of days expected to take, attachments of scanned (other form of softcopy) of relevant documents-see below). Among others
     NB: Copy as many people relevant people at the PT participants' side and provider as possible in the email.
   - Proof of delivery of notification can be either through (i) reply of acknowledgement email from the contact person or delegate (ii) proof of delivery by the courier company (iii) otherwise use date on result form.

   (c) Local participants (within the Uganda)
   - Follow SOP and guidelines available for TB specimen referral system already in place.
   - *Where applicable,* notify those participants' whose email addresses are available at NTRL and notify regional teams representatives both at NTRL and NTLP **one (01) week** before the shipment commences who are responsible for transfer of information downwards.

   (d) Delivered by PT provider directly to the participants
   - The PT items package is delivered by NTRL driver, Lab technician or any other designated NTRL employee.
   - Participant signs *"Form PT 013 F4 Acknowledgement of PT material receipt"*

6.2.2 Shipment or transport and distribution of PT material to participants

NB. All shipments of PT materials are door to door *unless otherwise stated*

A. For shipment out of Uganda

1. Put original copies of instruction to participants, results forms etc. inside the transport box.
2. Put original copies of shipment documents and instructions to participants in a separate envelope (*Don't put inside the transport box because they may be required for custom's clearance during transportation to the destination)*

3. Handover both the transport box and envelope of shipment of documents to courier company personnel and sign on the Courier company Pick Up Form (Keep a copy on File at NTRL) *NB. If courier company does not have pick up, then Use Form PT 013 F14. Acknowledgement of PT material receipt Form)*
4. Email scanned copies of shipment documents, Instructions to participants, results forms and others relevant to the contact person of participating lab.
5. The courier company sometimes sends e-mail acknowledgement of pick up from NTRL

B. For Shipments within the country

1. Put original copies of instruction to participants, results forms etc. inside the paper pouch of the Ziplock bag containing the PT material.
2. Transfer contents into a bigger envelope such as A4, Label the envelope with "Consignee details" and "FRAGILE" in bigger readable letters using a Marker or Print out labels.
3. Write all packages in the "POSTA PICK UP" register available in the main office space of the lab.
4. The POSTA courier personnel picks up all the packages and counter signs in the register acknowledging pick up for distribution to the respective destinations.
   OR
5. In case where the package is delivered by personnel from NTRL to the PT participant.
6. Document on *Form PT 013 F14. Acknowledgement of PT material receipt Form*

6.2.3 Stability and safety of PT material during transit

1. The preparation procedure of the panel and packaging is to ensure that the PT material is not comprised.
2. The PT material are put in/on transport material to ensure stability (and viable where applicable) during transit until the package reaches destination.
3. The PT materials have been in the safest way according to the safety level suitable to protect the shipper and the material.

6.2.4 Confirmation of receipt

1. For international PT materials, confirmation of receipt date may be through (a) email from participating lab (b) email from courier company with contacts of person receiving the package or from the courier company
2. For Local shipments through POSTA services, confirmation of receipt date maybe through (a) phone calls to the participating labs (b) Proof of delivery forms (POF) from POSTA services.
3. For deliveries done directly by an NTRL individual, confirmation of receipt date is on the *Form PT 013 F4. Acknowledgement of PT material receipt Form*

   NB: In instances where the PT provider can't determine proof of delivery, the date of delivery will be that on the results form during result analysis

6.3 In-case of loss or damage during transit

1. Expected time of transit to the destination is 14 days
2. However, if delay is pronounced beyond the maximum time (beyond 06 weeks) then the participant is advised not to test the PT material

3. Determine causes of the delay or loss of package to ascertain whether to send or not to send another package.
4. Prepare and send another PT to the participant *if possible*

7. References

External quality assessment for AFB smears microscopy, IUATLD

Protocol preparation of strains; Organization of rounds Quality assurance DST for WHO from Institute of Tropical Medicine; Belgium

R-8002 Proficiency Testing and other comparison programme requirements for testing and medical labs
