# Supplementary material for: Implementation of GeneXpert MTB/Rif proficiency testing program: A Case of the Uganda national tuberculosis reference laboratory/supranational reference laboratory
Source: PLoS One. 2021 May 14;16(5):e0251691. doi: 10.1371/journal.pone.0251691 (PMC8121318; doi:10.1371/journal.pone.0251691)
Supplement: S4 File — (PDF) [file pone.0251691.s007.pdf]

The Republic of Uganda

MINISTRY OF HEALTH

NATIONAL TUBERCULOSIS AND LEPROSY CONTROL PROGRAMME

Data analysis and evaluation of proficiency testing scheme results

SOP PT014 **Version 4.0** **Effective date:** **Initials authorizer:**

SOP Approval

	Name	Signature	Date
Prepared by			
Reviewed by			
Authorized by			
Date Retired:			

Approved changes

Brief description of the change

Annual Changes and Reviews

Name of reviser	Changes compared to previous version

1. Data analysis and evaluation of proficiency testing scheme results

2. Objectives and scope

This SOP describes how Uganda NTRL/SRL analyzes and evaluates data from proficiency testing schemes results, microscopy, culture and DST testing schemes. This SOP is applicable to all staff handling the PT scheme activities.

3. Abbreviations, definitions and terms

DST – Drug Susceptibility Testing
EQA – External Quality Assessment
IUATLD – International Union Against Tuberculosis Lund Disease
NTRL – National TB Reference Laboratory
PT – Proficiency testing
TAT – Turn Around Time

4. Tasks, responsibilities and accountabilities

Task	Responsible	Accountable
Data analysis and evaluation of PT scheme results.	All lab personnel	Lab manager

5. Safety and environment

N/A

6. Type of PT schemes

GeneXpert – Qualitative Nominal (*since the results are Categorical i.e MTB detected or MTB not detected and RIF Resistant detected or RIF Resistant not detected*

Procedure

1. In the event that a participating laboratory obtains results using a different test or measurement method, the data generated shall be analysed independently against an assigned/judicial value.
2. The choice of method/procedure by a participating lab shall not affect the assigned/judicial value.
3. A comparison of the overall outcome performance against the chosen methods/procedures shall also be done

6.1 Procedure of GeneXpert MTB/RIF® PT scheme data analysis

1. Receive peripheral results either by email or other means and ensure that a copy is saved on the server under the PT Name and round Number
2. Rename peripheral results as “Lab code” “PT name” “Peri” “date of receipt” e.g. 001 GXP Peri 20/04/2016 and on the hardcopy Record receipt date of PT results and technical personnel initials on the peripheral result form e.g received by NJ 2/6/2017
3. Record results and related information in the excel form PT 014 F11 GXP PT Data aggregation form (as illustrated in microscopy PT procedure 6.1.2 (6) steps (b) up to (k) above with slight changes).

a.

A	B	C	D	E	F	G	H	I	J	K	L	M	N	O
Code: PT 014 F11		Version 1.0		Effective date: 01-Mar-2017 Authorized by: Lab Manager										
Country	Lab name	Lab code	Date PT shipped	Text Date PT shipped	Date PT received	Text Date PT received	Shipping TAT	Date PT reported	Text Date PT reported	Closing date	Text closing date	Report issue	Text Report Issue date	Results TAT
Country	Lab name	Lab code	Date PT shipped	shipped	received	received	Shipping TAT	reported	reported	Closing date	date	Report issue	Issue date	Results TAT
Country X	Lab X	12	10/10/2017	10/17/2017	7	10/26/2017								
Country Y	Lab Y	13	10/10/2017	10/23/2017	13	10/24/2017								
	TOTAL	124			20									
	TAT AVERAGE				0	0								

b.

P	Q	R	S	T	U	V	W	X	Y	Z	AA	AB	AC	AD
												Percent sc	PASS or FAIL	Recommendation
Q1		Q2		Q3		Q4		Q5		Comment from participant	Comment	Percent score	PASS or FAIL	Recommendation
MTB Detected	Rif detecte	MTB Detected	Rif detected	MTB Detected	Rif detecte	MTB Detected	Rif detected	MTB Detected	Rif detected					
NEGATIVE		POSITIVE	RESISTANT	POSITIVE	SENSITIVE	POSITIVE			SENSITIVE					
NEGATIVE		POSITIVE	RESISTANT	POSITIVE	SENSITIVE	POSITIVE			SENSITIVE					

4. To create a worksheet to be used for “mail merging”, use data in sheet result received analysis PT 014 F2(B)
   (a) Right click on sheet copy PT 014 F2(B), select <move or copy>. Highlight <create a copy>, <move to end>. Press **OK.** This creates another copy at the end of the named sheets
   (b) Rename the created copy as “For mail merging-PT Round No”

Preparation of individual participant feedback report

1. See mail merge procedure below ‘’section 6.6”
2. Report is ready for sending as softcopy on email or hardcopy through a courier company. *(Proceed to SOP PT 015 Reporting PT scheme results)*

6.7 Procedure for calculation for performance statistics

Since the results for the different four PT schemes offered at NTRL **are qualitative results,** then the method/technique used for Calculating of performance statistics is **“to compare a participant's result with the assigned value.**

(a) If they are identical, then performance **is acceptable *and otherwise* as** described in conditions in 6.4 above.

6.8 Monitoring performance over time

Performance overtime will be monitored for (a) overall PT scheme for an individual laboratory.

(a) overall PT scheme. This performance will be monitored through the use of graphs in the PT report.
(b) individual laboratory; *where applicable* this will be monitored by comparing a participants' performance of the current with that of the previous round. This will be done only (*unless otherwise)* for participants with non-satisfactory performance in the current round of PT. This can be monitored using graphs or a statement in the PT report.

6.9 Procedure for demonstrating maintenance of proficiency test item homogeneity and stability

Since Uganda NTRL/SRL PT panels are either qualitative or semi quantitative, there are no defined statistical designs to be used (ISO 13528). However, NTRL as the PT provider has described processes for homogeneity and stability for each PT panels in SOP PT 009

8. References

- External quality assessment for AFB smear microscopy, IUATLD
- Protocol preparation of strains; Organization of rounds Quality assurance DST for WHO from Institute of Tropical Medicine; Belgium
- R-8002 Proficiency Testing and other comparison programme requirements for testing and medical labs
- 17043 standard
- Selection, use and interpretation of PT schemes Second Edition 2011 Eurachem PT Guide
- GeneXpert manual
- ISO 13528 Statistical methods for use in proficiency testing by interlaboratory comparisons First edition 2005-09-01
