# Supplementary material for: Implementation of GeneXpert MTB/Rif proficiency testing program: A Case of the Uganda national tuberculosis reference laboratory/supranational reference laboratory
Source: PLoS One. 2021 May 14;16(5):e0251691. doi: 10.1371/journal.pone.0251691 (PMC8121318; doi:10.1371/journal.pone.0251691)
Supplement: S5 File — (PDF) [file pone.0251691.s008.pdf]

UGANDA NATIONAL TUBERCULOSIS REFERENCE LABORATORY PROFICIENCY TESTING SCHEME

GeneXpert MTB/RIF® PROFICIENCY TESTING SCHEME PARTICIPANTS INSTRUCTION MANUAL

ROUND No: xxx

1.0 Introduction

The Uganda National Tuberculosis Reference Laboratory (NTRL) Proficiency testing scheme offers proficiency testing items to laboratories performing microscopy, line probe Assays, GeneXpert MTB/RIF ® assay, and drug susceptibility testing techniques. The design of the Uganda NTRL/SRL PT scheme includes randomly selected sub-samples from a bulk homogeneous supply of material which is distributed simultaneously to participating laboratories.

The NTRL management welcomes you to this year's PT round and wishes you good luck.

2.0 Proficiency test item (treatment and analysis)

The package contains 04 cryovials/isolates labelled Q1 to Q4. Examine and treat the specimens as you would for normal routine clinical specimens. Analyse the PT items using own internal SOPs.

3.0 Survey requirements

By accepting to participate in this PT scheme, your laboratory conforms to the all requirements of the PT scheme and ensuring that all minimum safety measures are used while handling the PT items. The management of the participating lab is required to provide the minimum safety guidelines and requirements and ensure that the PT items are ***NOT MANIPULATED*** unless the stated minimum safety requirements are in place.

Each participant laboratory must have a laboratory code sent together with the accompanying documents of this PT item. Please contact NTRL if unsure of your laboratory code.

4.0 Factors that may affect the testing of the PT items i.e. nature of PT items, storage conditions, whether the PT items are limited to selected methods and timing of the PT item,

Nature of PT items: the PT items are non-infectious since *Mycobacterium* isolates are non-viable. **Temperature;** the PT items are shipped and transported at ambient temperature. They should also be processed at ambient conditions.

Method/tests to be used; test following internal laboratory procedures. Keep the panels under refrigeration pending testing.

5.0 Source of PT items

The PT are prepared from routine materials available at NTRL. Standard control strains of *Mycobacterium tuberculosis (MTB)* H37R*v*, *Mycobacterium fortitium* well characterised strains from the Uganda NTRL pool of PT materials and Phosphate buffered saline (PBS) for the Negative panels.

6.0 **Selection of samples**
All participating laboratories receive identical PT items labelled Q1 to Q4. The PT items selected have varying degree of MTB identification and resistance pattern.

7.0 **Homogeneity and stability of PT items**
The PT provider has ensured that the PT items' homogeneity and stability is maintained. This has been done through internal quality control procedures.

8.0 **Panel details and sample preparation**
The PT panel package contains 04 cryovials each containing at least 01 mL of the PT item. Strain suspensions are prepared from culture, inactivated by autoclaving. Proof of inactivation is confirmed by negative growth on culture.

9.0 **Sample reception, special handling instructions and safety requirements**

9.1 Open the PT package **in a certified biosafety cabinet.**

9.2 Inspect for any breakages, possible deterioration during transit, correct PT items and labelling (total of 04 isolates), participant instruction manual, result reporting form and accompanying documents as stated.

9.3 Report to PT provider contacts in case of unsuitable testing Pt items within 5 days after receipt (*NB. The PT package is unsuitable for testing after 3 weeks in transit)*

9.4 Sub culture the PT items immediately NB. Don't decontaminate the PT items at this stage

9.5 Incase PT items are not processed immediately, store at refrigerator temperature 2-8°C for a maximum of 2 days from date of receipt

NB. In case of leakage, discard following national or international guidelines. Please follow local and international safety precautions at all times

10.0 **Specific environmental conditions that the tests must be performed at**
No specific conditions. Process and store PT items at room temperature. Only discard after final performance results are received from the PT provider.

11.0 **Sample dispatch notifications and actions to be taken if sample was not received, instructions on returning of proficiency test items**
Sample dispatch notifications are disseminated through email to contact(s) of the participants especially for international participants. Local participants are communicated using the existing local systems. Report to PT provider contacts in case of unsuitable testing Pt items within 5 days after receipt

12.0 **Instructions for PT analysis**

(i) Each PT panel should be handled as routine samples and each cryovial is to be tested on one module.

1.	Label four falcon tubes (or its equivalent) as Q1, Q2, Q3 and Q4 corresponding to the PT cryovial labels.

2.	Using a new sterile pasture pipette for each tube, transfer 01 mL of PT material into a pre-labelled falcon tube (or its equivalent).
3.	Add 2 mL of the GeneXpert MTB/RIF reagent buffer .
4.	Vortex/Shake the mixture vigorously for 5 seconds and allow to stand for 10 minutes
5.	Vortex/Shake the mixture vigorously for 5 seconds and allow to stand for more 5 minutes.
6.	Label four GeneXpert® MTB/RIF cartridges as Q1, Q2, Q3 and Q4 corresponding to the pre-labelled falcon tubes (or its equivalent) used.
7.	Using a pasture in GeneXpert box, transfer 2 mL of each sample mixture to the corresponding pre-labelled GeneXpert® MTB/RIF cartridge.
8.	Load the cartridges in the GeneXpert machine.
9.	After completing testing, fill in results on the *GeneXpert MTB/RIF® result form* provided

(ii) Ensure local and international laboratory safety procedures are follow

13.0 Instructions for completing the survey

The PT package contains 04 isolates labelled Q1 to Q4, use internal geneXpert procedure. Report results obtained using *attached GeneXpert PT scheme result form* .

14.0 Recording and reporting of PT panel results

- Return hardcopy or scanned *GeneXpert PT scheme result form* to the contact(s) below of the PT provider within 10 working days.
- The participants whose result are submitted by email should receive acknowledgement of receipt at most after 03 working days. Please notify NTRL if *no acknowledgement* is received.

15.0 Closing date for submission of results and consequence of late submission of late submission or no returns

**EQA panels category**	**GeneXpert**
Frequency/ year	Biannual
Sending out Month	February and August
Quantity	04 isolates/round
Expected TAT (from date of dispatch)	44 days

Late submission: All results after the Final PT scheme report will not be accepted *unless otherwise*

16.0 Evaluation of submitted results, reporting

- The assigned value is determined by the PT provider i.e. NTRL during preparation of the PT panel

- For PT item for which participant does not provide a result, the PT item is not included in the evaluation and analysis of the PT scheme. It is labelled as “Not Applicable or N/A”
- Errors and scoring system is as follows; each module shall be scored as **PASS** or **FAIL**. 10 and 5 shall be used as the scores. A module shall be scored 10 if it passes both detection of MTB and RIF resistance, and scored 5 if it fails detection of either MTB or RIF resistance. To pass a module test, 10 score is expected. Modules which report; errors, no result or invalid shall not be scored.

- For the participant to pass the microscopy PT, the total score of the 4 PT items should be at least 80%.

17.0 **Sub contracted PT activities**

Only PT package courier services are sub contracted.

18.0 **Corrective action and root cause analysis**

Each participating laboratory which does not score the maximum possible points is strongly to perform an internal root cause analysis. Although, it is mandatory to provide the results of the root cause analysis to the PT provider, the PT provider is willing to work with the participants during this process.

19.0 **Disputes and appeals**

Each participant contact person should provide dispute of the results within five (05) working days after receipt of the PT results. The NTRL investigates the disputes and provides feedback within two (02) from the date of PT results receipt by the participant.

Contact details

The name and address of the proficiency testing provider;

National TB Reference Laboratory Uganda
P.o. Box 16041, Kampala Uganda
Plot 106-1062, Butabika Road, Luzira.
Opposite Butabika Hospital – Kampala, Uganda.
Toll free: 0800111133
Email: pt@ntrl.or.ug
