# Supplementary material for: Implementation of GeneXpert MTB/Rif proficiency testing program: A Case of the Uganda national tuberculosis reference laboratory/supranational reference laboratory
Source: PLoS One. 2021 May 14;16(5):e0251691. doi: 10.1371/journal.pone.0251691 (PMC8121318; doi:10.1371/journal.pone.0251691)
Supplement: Attachment — Submitted filename: response to reviewers.docx [file pone.0251691.s009.docx]

PlosOne Editorial committee

17^th^/November/2020

Dear Sir/Madam

I extend my appreciation to the precious time and dedication given to my manuscript. The reviews and comments raised have been taken positively and as authors of this manuscript, we would like to respond to them as below;

**Review Comment**: It is noted that the text has similarity to atleast one other study PMID : 30567748. So, the text must also be revised for originality

***Response:*** *The manuscript has been exclusively revised to exhibit originality of research and bring out the innovation of using liquid panels for GeneXpert proficiency Testing panels with clear distinction from the research work presented in study PMID : 30567748*

**Response to Reviewer 1**

1. What is the reason behind author’s choice for using autoclaving as an inactivation method when SR reagent is a proven method of inactivation for Mtb? Did the authors conduct any studies to verify that autoclaving does not impact the GeneXpert results?

*Yes, we conducted a preliminary experiment by inactivating eight samples using either method and inoculating on both LJ culture and MGIT culture after inactivation. This was done at the initiation of this study. The preliminary results obtained from inactivation of samples for GeneXpert proficiency testing, showed that the method of using sample reagent (SR) buffer to inactivate samples yielded less effectiveness than the autoclaving method. Proof of inactivation results gave two sample strains inactivated using sample reagent (SR) showing growth on both MGIT method and LJ culture method at the 5^th^ week of culturing while all those inactivated using autoclaving method showed no growth.*

2. Further, authors note that samples are stored at 2-8C for 42 days until results from MGIT are verified for inactivation. Does this long storage of samples have any impact on the results? Were any experiments conducted?

*Yes, experiments were done to ensure stability and consistency. For every round prepared, pretest of five runs on inactivated sample was done before storage at 2-8°C and after the forty two days a validation test was done to prove consistency of M.tb DNA in the samples as highlighted on line 162-164 and 169-171. The results showed no difference on the expected results.*

3. It is noted in the supplementary section that samples are shipped at ambient temperature. Were overnight shipment methods used? What was the longest it took for samples to arrive at a designated laboratory? Was there any correlation with long transit times and discordant/invalid results? In other words, did long transit times lead to sample degradation and hence invalid results?

*In this study no overnight shipment methods were used. The longest it took samples to reach final destination was fourteen days. The time taken to receive samples in this study showed no correlation with obtaining discordant results as highlighted in line 371-374. To ascertain whether our samples were still stable and consistent even after shipment were performed a stability test on a set of panel for each round. This panel was kept at ambient temperature and tested after three weeks from date of dispatch as shown in line 193-198.*

4. Authors are requested to provide an original reference for lines 110-111 for the detection limit of GeneXpert MTB/RIF test

*This has been added on line 111 (citation 21; Study done by Danica Helb et, al in 2010*

5. Under supporting information, authors listed figures 1 to 5 as S1 Fig 1, S2 Fig 2 etc., Please clarify which figures belong in the main manuscript and supplementary sections respectively.

*Figures 1-5 all belong to the main manuscript and have been added.*

6. There is no information about the process behind obtaining ISO accreditation. Some detail about what changes were made to the SOPS to align with ISO’s requirements and how this aided in study’s success supporting lines 438-439 would be helpful

*Line 120-134 have been added in methods to detail the process behind ISO 17043 accreditation. Line 402-407 have been added also to show the impact of aligning to ISO 17043.*

7. Authors note that the major cause of false results is clerical errors. Did the authors consider using automated GeneXpert test reports instead of manual reporting?

*During this study, we noticed the need to enroll an electronic system but we were unable to enroll it out during the study period. The system has developed in 2020 and piloted in Uganda but not yet put to full use.*

8. Were the panel of strains freshly prepared during each around? Please clarify in methods section.

*Panels were freshly prepared for each round as highlighted on line 185-187*.

9. How are the results reported when a test reports no MTB detected but RIF sensitivity/resistance is detected?

*The score was zero (0) for a site that reported “no MTB detected but RIF sensitivity/resistance is detected”. This being that rifampicin resistance could not be expected without MTB detection.*

10. Minor comments

All acronyms have been expanded the first time they appear in the manuscript. For consistency acronym PT has been used in the entire manuscript. The entire manuscript has been revised to address grammatical errors.

**Response to Reviewer 2**

1) EQA should be defined as it is its first use.

*This has been addressed.*

2) The scoring system

*For the scoring system, we have added the fraction score, e.g. 6 out of 8 points (line 262-266) in the methodology write up as suggested by the reviewer to clarify on the passing threshold. The adoption of a percentage score was to align the participant reports to the requirements of ISO 17043 clauses on data analysis that required percentage-scoring system.*

3) Names of the study rounds

*The round number have been renamed as; Pilot study retained its naming, round 2 2016 to* ***round 1 (2016)****, round 1 2017 to* ***round 2 (2017)****, round 2 2017 to* ***round 3 (2017),*** *and round 1 2018 to* ***round 4 (2018).***

4) Result discrepancy

*The results have been thoroughly re-analyzed from the raw data to address the discrepancy in the earlier submission as noticed by the reviewer.*

**6a) The results in table 1 for pilot study were corrected to,**

| *parameter* | *Before review* | **After review** |
| --- | --- | --- |
| *Total results returned* | *48* | **51** |
| *No returns* | *4* | **1** |
| *Non-functional sites* | *3* | **3** |

**In table 2, the results remained as before for the pilot study as follows**

| *parameter* | *Before review* | **After review** |
| --- | --- | --- |
| *Satisfactory score* | *48* | **48** |
| *Unsatisfactory score* | *3* | **3** |
| *Non-functional sites* | *3* | **3** |

**In table 1, the results remained as before review for round 3 (2017)**

**The results in table 2 for round 3 (2017) were corrected as below,**

| *parameter* | *Before review* | **After review** |
| --- | --- | --- |
| *Satisfactory score* | *100* | **99** |
| *Unsatisfactory score* | *5* | **5** |

**In table 1, the results remained as before review for round 4 (2018)**

**The results in table 2 for round 3 2017 were corrected as below,**

| parameter | Before review | **After review** |
| --- | --- | --- |
| Satisfactory score | 115(97.5%) | **128(95.5%)** |
| Unsatisfactory score | 4(2.5%) | **6(4.5%)** |

*Line 297 (formerly line 288), number of times with sites scoring unsatisfactory was adjusted to 32 to match results in table 2 after data re-analysis.*

*Table 3 and Table 4 were added to show the number of laboratories scoring satisfactory and those with unsatisfactory respectively with their continuous times of participation.*

*Figure 4 graph has been added after re-analysis to show probability of scoring satisfactorily given the number of times a laboratory consistently participates*

*Table 5 was added showing to breakdown the different discordant results (false positives/negatives/resistant/sensitive)*

5) List of issues raised during on and off site follow up

*Table 6 has been added to show the frequency of issues raised during on and off site follow up*

6) Performance of a laboratory inside or outside Uganda

*The performance of laboratories within and outside Uganda had no significant difference and scores were seen not to vary at all in regards to the location of the laboratory as highlighted in line 446-448.*

I would like again to appreciate the time awarded for this manuscript.

Thanks PlosOne

Yours faithfully

Kabugo Joel;

Author **Implementation of GeneXpert MTB/Rif proficiency testing program: A Case of the Uganda national tuberculosis reference laboratory/Supranational reference laboratory**
