# Supplementary material for: Implementation of GeneXpert MTB/Rif proficiency testing program: A Case of the Uganda national tuberculosis reference laboratory/supranational reference laboratory
Source: PLoS One. 2021 May 14;16(5):e0251691. doi: 10.1371/journal.pone.0251691 (PMC8121318; doi:10.1371/journal.pone.0251691)
Supplement: Attachment — Submitted filename: Response to Reviewers.docx [file pone.0251691.s011.docx]

PlosOne Editorial committee

24^th^/April/2021

Dear Sir/Madam

I continue to extend my appreciation to the time given to my manuscript and below is my response to the reviews made;

### Content Review

Line 55- ' mycobacterium tuberculsosis ' has been corrected to 'Mycobacterium tuberculosis'

Line 165 and 166- ' mgit and lj' has been re-written as MGIT and LJ and also expanded.

Line 421- were has been replaced by 'where'

## References review

Reference (2) has been re-referenced citing it as it is supposed to be written. Earlier in the manuscript, its citation was incomplete.

Reference (Agizew T, Boyd R, Ndwapi N, Auld A, Basotli J, Nyirenda S, et al. Peripheral clinic versus centralized laboratory-based Xpert MTB/RIF performance: Experience gained from a pragmatic, stepped-wedge trial in Botswana. PLoS One. 2017 Aug 1;12(8).) formerly reference (11) in the manuscript has been removed from the manuscript. This is because the information it carries does not strongly connect with the content matter mentioned in line 67-68.

Reference 13 has been re-referenced. Earlier in the manuscript, its citation was incomplete

Reference 18 has been re-referenced. Earlier in the manuscript, its citation was incomplete

Reference 21 has been re-referenced. Earlier in the manuscript, its citation was incomplete.

All the other references have been crosschecked for the correctness and support of content in the manuscript and we feel they are fit to be cited.

I once again appreciate the time awarded for this manuscript. Thanks PlosOne

Yours faithfully

Kabugo Joel; Author **Implementation of GeneXpert MTB/Rif proficiency testing program: A Case of the Uganda national tuberculosis reference laboratory/Supranational reference laboratory**
